# Mental Health Among Informal Caregivers of Older Adults in Asia

**DOI:** 10.1093/geroni/igab046.938

**Published:** 2021-12-17

**Authors:** Xiang Gao, Kaipeng Wang, Fei Sun

## Abstract

The purpose of this symposium is to highlight the mental health needs and factors associated with mental health among informal caregivers of older adults in Asia. The symposium consists of five papers. The first paper explores the perceived role, needs, and rewards of informal caregiving among caregivers of residents in independent long-term care facilities in South India. The second paper presents a systematic review and meta-analysis on the association between long-term care service use and informal caregiver burden, depression, and health status. The third paper examines the association between caregivers’ characteristics and quality of life among informal caregivers of older adults with cognitive impairment in China. The fourth paper examines the association between coping strategies and caregiver burden and depression among Chinese caregivers of older adults with cognitive impairment. The last paper examines the association between cohort, meaning making, and depression among adult caregivers during the COVID-19 pandemic in Hong Kong. Taken together, these five papers underscore of the mental health needs and protective and risk factors of mental well-being among caregivers in Asia. Findings of those papers inform the development and adaptation of culturally sensitive interventions to improve mental health outcomes among informal caregivers in Asia. The disccuant will comment on the strengths and limitations of these papers in terms of their contributions to the theory, research, and practice on mental health among informal caregivers in Asia.

